# Relation between Attachment and Obesity in Preschool Years: A Systematic Review of the Literature

**DOI:** 10.3390/nu13103572

**Published:** 2021-10-12

**Authors:** Ana F. Santos, Mariana C. Martins, Carla Fernandes, Kelly K. Bost, Manuela Veríssimo

**Affiliations:** 1 William James Center for Research, ISPA—Instituto Universitário, 1149-041 Lisboa, Portugal; ana.f.santos2@hotmail.com (A.F.S.); mariana.g.c.martins@hotmail.com (M.C.M.); csfernandes@ispa.pt (C.F.); 2 Human Development and Family Studies, University of Illinois, Champaign, IL 61801, USA; kbost@illinois.edu

**Keywords:** attachment, obesity, systematic review, preschool

## Abstract

Increasing evidence suggests that attachment plays an important role in obesity. However, few studies examined this relationship in preschool children. This study aimed to systematically examine the empirical, peer-reviewed evidence regarding the relationship between attachment quality and obesity in the preschool years. Using established guidelines, relevant peer-reviewed literature published between 2000 and July 2021 was searched through EBSCO. This yielded a total of 1124 records for review. Established inclusion criteria comprised: empirical studies published in peer-review journals; include at least one anthropometric measure and/or food consumption measure. Exclusion criteria comprised: attachment measures not following Bowlby-Ainsworth conceptualization of the construct; children in institutionalized settings; context of severe mental illness, documented substance use disorders, or eating disorders; include only a measure of the psychological aspects of eating; intervention programs. After exclusions, eight studies with a total of 9225 participants met the inclusion criteria. Results support the role of attachment in weight-related outcomes, suggesting that considering attachment in the risk of obesity could contribute to the elaboration of effective prevention and intervention programs. Limitations included the small number of studies, predominately cross-sectional designs, the diversity of methodologies, most samples not including fathers, and lack of evidence about the developmental mechanisms underlying the association between attachment and obesity. More evidence is needed to determine how attachment and obesity are linked, and the potential underlying mechanisms accounting for this relationship.

## 1. Introduction

Childhood obesity has reached alarming proportions globally, affecting every country in the world. It is considered one of the most serious public health challenges of the 21st century and it has been described as a “ticking time bomb” [1]. The most recent data show that 39 million children under the age of 5 were overweight or obese in 2020 [2]. Obesity is not only a chronic disease in itself, but also a major risk factor for cardiometabolic and psychosocial problems [3,4,5], premature death [6,7] and it has a major adverse impact on the economy and society [8,9]. Obesity in childhood is especially worrisome because overweight or obese children are at risk of remaining overweight or obese in adulthood [10,11].

Given these findings, there is an urgent need to document obesity risk factors in early childhood that can contribute to the development of effective prevention and intervention strategies. In fact, childhood obesity has revealed to be very difficult to prevent and treat because of its complex etiology [12,13]. Factors such as genetic variants (e.g., [14,15]) physical activity and sedentary living (e.g., [16,17]) and food availability (e.g., [18,19]) have been the main focus of obesity research, however childhood obesity can only be partially explained by these factors [20,21]. On the other hand, little attention has been given to developmental and relational influences on the etiology of obesity [22].

A growing body of research suggests that the emotional quality of relationships between caregivers and young children play an important role in childhood obesity (e.g., [23,24,25,26,27,28,29]). In a meta-analytic review, insecure attachment relationships were positively associated with a higher body mass index in both children and adults [30]. Possible explanations of this association involve poor emotion regulation and heightened psychophysiological responsiveness to stress. Previous research has shown that difficulties in emotion regulation, which result in maladaptive physiologic and behavioral responses to stress, are associated with obesity [31,32,33]. In fact, there is a large body of evidence connecting emotions to eating behaviors suggesting that emotion dysregulation is a risk factor for eating beyond satiation [34,35], particularly high-energy foods [36,37,38].

Attachment theory is one of the most comprehensive developmental theories for understanding human development, especially socioemotional development [39]. Attachment security is inextricably linked to experiencing, regulating, and coping with stress [40]. The attachment bond between a child and their attachment figures functions as a regulatory system because caregivers act as secure base whom a child leaves to explore and to whom he/she returns as a safe haven when distressed [39,41,42,43,44]. Based on their attachment histories (i.e., attachment figures’ history of success in providing comfort and protection in times of distress), children develop expectations about how their attachment figures will respond to future expressions of distress and form strategies hinged on these expectations to seek comfort from their attachment figures [45,46]. Consequently, different patterns of emotion regulation can emerge from attachment histories [40,47,48].

Attachment security is formed when caregivers are consistently available, sensitive, and responsive to their children’ emotional states and needs [49]. Securely attached children are able to use their caregivers as a secure base from where they can explore their world because they formed expectations that they will consistently receive support and protection when needed [39,50,51,52]. In other words, secure children have learned that when potential threats arise, caregivers will be accessible and supportive, bringing the emotional comfort and distress relief [53,54]. For that reason, secure children generally use adaptive emotion regulation strategies, which involve open, direct, and active expression of emotions to the attachment figure [52,55]. Conversely, when caregivers are rejecting or inconsistent in their availability and response to signals and cues of distress, other emotion regulation strategies develop [56]. In case of an unresponsive attachment figure (i.e., a caregiver who is unlikely to respond to the child’s pronounced signals of distress), a deactivating strategy may develop. Children learn to minimize or hide their distress in order to avoid the anticipated painful consequence of expressing their negative emotions [55,57,58]. In case of an inconsistent attachment figure, a hyperactivating strategy may develop. Children learn to maximize their distress in an effort to increase the probability of gaining the attention and support from their caregivers [45,55,58]. Insecure children are then more likely to use maladaptive emotion regulation strategies, which either heighten or supress distress instead of effectively ending it [54,55]. As children grow, these early childhood interactions with attachment figures are internalized in enduring beliefs and expectations about the self and others [39,49,59]. These representations are called internal working models and are believed to be the cognitive schemas by which early attachment experiences are sustained into adulthood [59]. As a result, one-way insecure children and adults may deal with stress and regulate their emotions is turning to eating [30,54]. Eating produces feelings of pleasure and reward [60], and high-energy foods have a calming effect on brain areas involved in stress response (e.g., [61]), such as the effect of satisfying interactions with significant others [30,62]. Therefore, eating can become a conditioned way to regulate physiological stress and negative emotions, making the discomfort disappear even if only momentarily [25,54,63].

Given the importance of early attachment relationships for the development of emotion regulation [53,54,64,65], attachment theory provides a consistent framework for understanding the relationship between emotion dysregulation and obesity. It is then possible that the caregiver–child attachment might contribute to childhood obesity-related outcomes by influencing children’s emotion regulation [28,66]. Furthermore, as caregivers are powerful socialization agents of children’s emotions [67], caregiver attachment might also play a role in childhood obesity because attachment insecurity is associated with the use of maladaptive emotion regulation strategies [68,69]. As a result, insecure caregivers may inadvertently socialize their children to minimize or maximize their distress [66]. Moreover, the organization of caregiver attachment is related to the security of caregiver–child attachment [51,70]. Therefore, considering both the caregiver attachment and caregiver–child attachment in pediatric obesity literature is important.

Research on the role of attachment in the etiology of childhood obesity has proliferated in recent years, with studies showing that both an insecure caregiver attachment (e.g., [28,71,72]) and an insecure caregiver–child attachment (e.g., [23,24,25,26,27,29]) might contribute to children’s overweight or obesity development. However, very few studies have focused on the preschool years, which are critical for the development of emotion regulation because children are becoming more autonomous in their regulation of emotions [73,74]. Therefore, the purpose of the current study was to systematically review the literature on the association between attachment quality (both caregiver attachment and caregiver–child attachment) and obesity (defined through anthropometric measurements and food consumption) during the preschool years. We expected positive associations between insecure attachment and a higher weight status or unhealthy food consumption and, conversely, negative associations between secure attachment and a higher weight status or unhealthy food consumption.

## 2. Materials and Methods

### 2.1. Eligibility Criteria

A set of inclusion and exclusion criteria were established for article inclusion. For abstract screening, the following inclusion criteria were established a priori: (1) empirical articles with available abstract published in peer-review journals; (2) articles published in Portuguese, English, French, or Spanish (languages mastered by the authors); (3) articles examining the relationship between attachment and obesity in preschool children; and (4) articles including at least one anthropometric measure and/or one food consumption measure. Exclusion criteria were established a posteriori: (1) studies measuring attachment that did not follow Bowlby [39] or Ainsworth and colleagues [49] conceptualization of the construct (2) children in institutionalized settings; (3) studies of attachment in the context of severe mental illness, documented substance use disorders, or eating disorders (e.g., anorexia, bulimia); (4) studies including only a measure of the psychological aspects of eating (e.g., emotional eating, loss of control over eating, disinhibited eating) instead of an anthropometric measure or a food consumption measure; (5) studies including only clinical samples; (6) intervention programs; (7) articles aiming to develop, adapt, or validate measures of attachment; and (8) studies with a qualitative design.

### 2.2. Search Strategy

This review was conducted in accordance with the guidelines from the Preferred Reporting Items for Systematic Reviews and Meta-Analyses (PRISMA) [75] and from the International Prospective Register of Systematic Reviews (PROSPERO) registration system to ensure the review process transparency, as well to improve the quality and confidence in findings. Review protocol was registered before data extraction on the PROSPERO (Registration Number: CRD42021247229).

A systematic data search was performed in EBSCO, via PsycINFO, Web of Science, PubMED, Scopus, and Psychology and Behavioral Sciences Collection using the following search terms (combined with Boolean terms): “attachment” AND (“obesity” OR “overweight” OR “weight” OR “adiposity” OR “BMI” OR “body mass index” OR “waist-to-hip ratio” OR “waist circumference” OR “body fat percentage” OR “food consumption”) AND (“preschool” OR “early childhood” OR “child” OR “toddler” OR “infant” OR “baby”). This search was applied to the last 20 years (until 17 July 2021).

### 2.3. Study Selection

**Figure 1:**
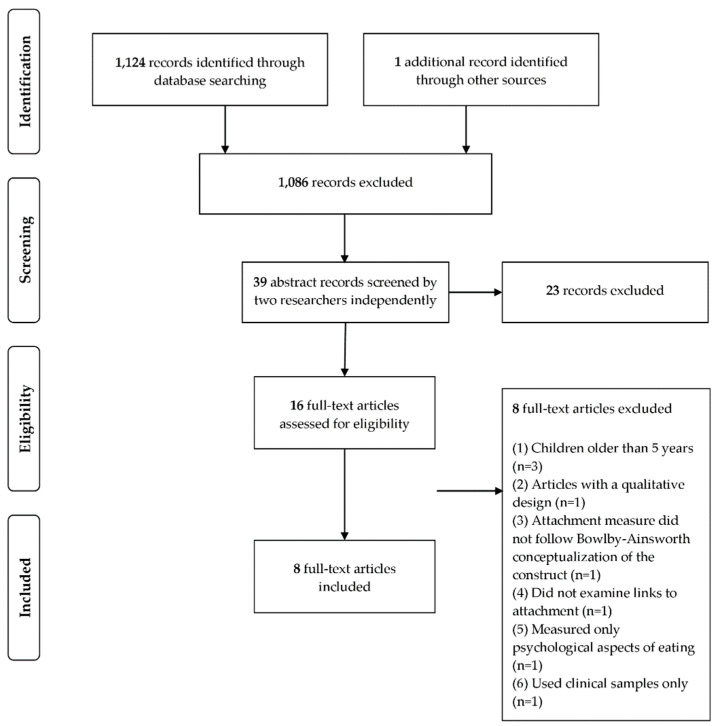
Flow diagram depicting study identification and selection process.

The systematic data search yielded a total of 1124 records. The initial 1124 articles were screened according to the established selection criteria by the first author and 1086 articles were excluded at this stage. The remaining 39 articles were screened by the first and second authors to assess eligibility for inclusion according to the established criteria, and 16 full-texts were further assessed independently by the first two authors for inclusion. Any discrepancy in inclusion/exclusion decisions was resolved through consensus. After the full-text review by the first two authors, a total of 8 articles met all the inclusion criteria (Figure 1).

### 2.4. Data Extraction

The data extraction protocol is in accordance with PRISMA guidelines [75]. To summarize the results, a categorization system was created. System categories included: (1) general characteristics of the studies (e.g., study design, sample size); (2) general characteristics of studies’ participants (e.g., ethnicity, age range); (3) assessments of attachment; and (4) assessments of obesity. The classification of the retrieved articles was performed by the first two authors. Disagreements were discussed until consensus was reached.

## 3. Results

### 3.1. Studies Characteristics

Table 1 provides a summary of each of the studies included in the review. The eight selected studies were published between 2010 and 2021. Studies were conducted in the United States of America (n = 4), Italy (n = 1), Germany (n = 2), and Sweden (n = 1). Five studies were cross-sectional and three studies were longitudinal.

Across studies, the total number of participants showed great variability, with a sample size ranging from 51 [76] to 6650 participants [24]. Regarding the participating children’s gender, four studies included more girls than boys, two studies included more boys, and two studies did not report on gender. Furthermore, the large majority of studies sampled only mothers (75%) and two studies’ samples included both mothers and fathers. No study sampled only fathers, or other caregivers.

Three studies’ samples were majority White (≥50%), one Latino (95%) and four did not report racial/ethnic demographics. The studies that did not report racial/ethnic demographics were from Europe. Of the four US studies, all reported on race/ethnicity. Considering socioeconomic characteristics, most participating caregivers had a high school degree or a college degree. Regarding the caregivers’ professional situation, only two studies refer this information [72,77], revealing that most caregivers were employed. Five studies reported on caregivers’ marital status or living arrangements, demonstrating that most children’s caregivers were married or cohabitating; however, in one of these five studies [77] it was not clear whether mothers were married/cohabitating with the child’s father.

Four studies [28,72,76,77] examined adult attachment, three studies [24,25,29] examined caregiver–child attachment and one study [78] assessed both adult attachment and caregiver–child attachment. The studies used psychometrically valid measures to assess attachment, conceptualized as either dimensional scores of attachment (n = 8), or categorical, in terms of a specific attachment style (n = 2). Two studies used both dimension and categorical assessment of attachment. Seven different measures of attachment were used across the eight studies. Two studies [28,72] used the Relationship Questionnaire (RQ) [59]. Two studies [76,78] used the Reflective Functioning Scale (RF-S) [79] with application to the Adult Attachment Interview (AAI) [80]. One study [77] used the Adult Attachment Scale (AAS) [81] and a slightly adapted version of the Experiences in Close Relationships Scale (ECR) [82], where the original 7-point response scale was reduced to a 5-point scale (1 = strongly disagree to 5 = strongly agree). Three studies [25,29,78] used the Attachment Q-Set (AQS) [83] and one study [24] used the Toddler Attachment Sort-45 (TAS-45) [84], a modified version of the AQS. Finally, one study [25] used the Strange Situation Procedure (SSP) [49].

Most studies (87.5%) assessed children’s weight status using anthropometric measures, and only one study [28] measured children’s food consumption with a self-report measure: the parent interview child health section from the Early Childhood Longitudinal Study-B (ECLS-B) [85]. Studies which examined children’s weight status (n = 7) relied mostly on BMI status (87.7%), with one study [77] relying on weight-for-length. In five studies, BMI was calculated based on measured weight and height [24,25,29,72,78]. In two studies, BMI [76] and weight-for-length [77] were calculated based on children’s weight and height reported by parents. To determine children’s weight status and regarding growth references, five studies calculated BMI percentiles, of which two [24,25] used the Centers for Disease Control and Prevention (CDC) [86] growth references, one [72] used the International Obesity Task Force (IOTF) [87] growth references, and two [29,78] did not report on how BMI percentiles were calculated and the growth references used. In one study [76] it was not discussed whether BMI percentiles or Z-scores were used. Lastly, in the study that used children’s weight-for-length scores, Z-scores were calculated based on World Health Organization (WHO) [88] growth references.

### 3.2. Overview of Key Findings on the Relationship between Caregiver Attachment and Obesity

Five studies which examined the role of parental attachment in children’s weight status and food consumption found that the caregiver’s insecure attachment is a risk factor for childhood obesity. Table 2 displays a summary of the studies’ key findings.

The study of Hepworth and colleagues [77] demonstrated that maternal attachment style according to the AAS predicted children’s weight-for-length Z-scores (r = −0.29; *p* < 0.05), such that mothers who classified themselves as secure had children with lower weight-for-length Z-scores compared to insecure mothers (avoidant or anxious). Neither maternal attachment avoidance nor attachment anxiety according to the ECR significantly predicted children’s weight-for-length Z-scores. Path analysis showed a significant direct effect of maternal attachment according to the AAS on infants’ weight-for-length Z-scores (c′ = −0.68, SE = 0.22, t = −3.05, *p* = < 0.01). In this study, maternal sensitivity was also assessed. An indirect effect of maternal attachment style on infants’ weight-for-length Z-scores via maternal sensitivity was also tested, indicating that this association was not mediated by maternal sensitivity (ab = 0.10, 95% CI = [−0.04, 0.31]). Although maternal sensitivity did not mediate the association between maternal attachment and infant weight-for-length, higher ratings of maternal sensitivity predicted higher infant weight-for-length.

Stenhammar and collaborators [72] analysed whether family stress and parental attachment style were associated with children’s BMI. The results indicated that insecure attachment in both mothers and fathers was associated with overweight in children, independently of confounding factors, but not maternal stress. Adjustment for maternal stress eliminated the associations with attachment style. Moreover, mothers’, but not fathers’, stress score was significantly associated with overweight and underweight in children, with adjusted odds ratios (and 95% confidence interval) of 4.61 (3.11–6.84; *p* < 0.01) and 3.08 (1.64–5.81; *p* < 0.01).

Two studies examined the specific role of maternal mentalization, assessed with the AAI-RF, on children’s BMI. In the study conducted by de Campora and colleagues [76], the association between maternal mentalization (assessed during pregnancy) and children’s BMI (at 3 years of age) was only marginally significant (r = −2.96; *p* < 0.10). In this study, mothers’ difficulties in emotion regulation strategies were also assessed during pregnancy with the Difficulties in Emotion Regulation Scale (DERS) [89]. When the authors added the AAI-RF, in addition to the DERS, to explain the variance of the children’s BMI, multiple regression models improved, with the effect sizes of the βweight being in the medium range. Multiple regression models revealed a trend suggesting that maternal mentalization might explain variance in children’s weight beyond the effects of maternal emotional dysregulation. Keitel-Korndörfer [78] also examined mother–child attachment. The results showed that there was an indirect influence of maternal mentalization on children’s weight, through mother–child attachment (90% CI [−3.559; −0.235]). Maternal mentalization was found to have a significant positive effect on mother–child attachment (B = 0.053, SE = 0.022, *p* < 0.01, one-tailed), and mother–child attachment a negative effect on children’s BMI percentiles (B = −27.160, SE = 14.631, *p* < 0.05, one-tailed), revealing that the higher the maternal mentalization, the higher the quality of mother–child attachment, and the higher the quality of mother–child attachment, the lower the child’s BMI percentile.

Only one study focused on food consumption. Bost and collaborators [28] explored the associations between caregivers’ attachment, caregivers’ emotion regulation and children’s food consumption. Path analysis showed that insecure caregivers were more likely to use ineffective emotion regulation, that is, to respond to their children’s distress in punishing or minimizing ways, and these negative responses increased caregivers’ use of emotion-related and pressuring feeding practices which, in turn, predicted children’s consumption of unhealthful foods.

### 3.3. Overview of Key Findings on the Relationship between Caregiver–Child Attachment and Obesity

The four studies that examined the role of caregiver–child attachment on children’s weight status also revealed evidence supporting this relationship. Table 3 provides a summary of the studies’ key findings.

Anderson and Whitaker [24] found that children with insecure attachment to mothers (measured at 24 months) were 1.30 times (95% CI [1.05; 1.62]) more likely to be obese at 4^1^⁄_2_ years of age than children with secure attachment, after controlling for several confounding factors (Table 3).

Likewise, Anderson and colleagues [25] demonstrated that insecure mother–child attachment at 24 months of age (assessed using AQS) was associated with increased odds for adolescent obesity, but not at 15 or 36 months (assessed using SSP). Additionally, at 24 and 36 months, the combination of insecure mother–child attachment and low maternal sensitivity was associated with greater odds of adolescent obesity than either was alone. Results also revealed that low maternal sensitivity was more strongly related to adolescent obesity than insecure mother–child attachment.

In the study of Keitel-Korndörfer and collaborators [29], univariate linear regression analyses showed a significant effect of the mother–child attachment on children’s BMI percentiles (B = −26.44, SE B = 13.59, β = −0.24, *p* = 0.03, one-tailed, R2 = 0.06), meaning that the lower the quality of the mother–child attachment, the higher the child’s BMI percentile. Multiple linear regression analyses showed that mother–child attachment predicted children’s BMI percentiles beyond biological parameters and mother’s marital status (Table 3).

## 4. Discussion

The aim of the present study was to systematically review the literature pertaining to the association between attachment and obesity in preschool years. Across the eight identified studies, we found evidence that supports the role of attachment in children’s weight-related outcomes. Insecure attachment (both caregivers’ attachment and caregiver–child attachment quality) was not only more prevalent in children with higher weight status (e.g., [29,77]), but was also a significant predictor of future weight gain (e.g., [24,25]). Moreover, this relation was found beyond the influence of other important biological and environmental factors implicated in the risk of obesity (e.g., [90,91,92]), such as caregivers’ weight status, level of education, professional situation, marital status, family income, and child’s birth weight status. These findings have important implications for the study of pediatric obesity and for the development of effective preventions and interventions.

In this review, we did not consider only children’s weight status to assess obesity, but also children’s food consumption. This decision was taken because the literature between attachment and obesity suggests that these two might be related through emotion regulation, a significant factor linking stress to obesity. In this sense, studies have found that stress and emotion dysregulation are related to increased consumption of between-meal snacks [93], higher sweet and salty food intake, and reduced lower calorie nutritious food intake such as fruits and vegetables, resulting in increases in children’s weight status [36,37,94]. For that reason, we believe that it is important to address not only the link between attachment and weight status but how attachment could influence eating which, in turn, results in differences in children’s weight status. However, we identified only one study assessing the relationship between attachment and food consumption in preschool years. Previous studies attempted to examine the relationship between attachment and eating behaviours, nevertheless the focus was on disordered eating [54]. Consequently, very few studies explored the relationship between attachment and eating behaviours or diet quality in non-clinical samples, especially in children [95]. In this sense, the work of Bost and colleagues [28] was the only one identified exploring this relationship in preschool years. This study is also important because it provided evidence for a possible mechanism underlying the association between attachment and children’s food consumption: emotion regulation. In fact, the literature on the relationship between attachment and obesity in both adults and children proposes that this association can be explained by the impact of attachment relationships on the capacity to regulate emotions and stress responses. More specific, it is suggested that insecure attachment might lead to ineffective emotion regulation, which in turn results in maladaptive psychophysiological responsiveness to stress [24,30,66].

Attachment histories result in individual differences in emotion regulation and in dealing with stress, because they are related to caregivers’ responsiveness and secure base behaviors [55]. When exposed to a threat or overwhelming challenge, children seek proximity to caregivers and their responsiveness can successfully alleviate children’s distress [47]. Under this condition, children can learn to regulate their negative emotions in adaptive ways [55]. In contrast, when caregivers are unresponsive to their children’s signals and cues of distress, children cannot use them as secure base and as a haven of security and therefore miss opportunities to experience intense emotions and practice their regulation in a safe manner [53]. As a result, children may develop dysregulated stress responses [54,55]. However, there’s a lack of evidence supporting emotion regulation processes and physiological responses to stress as underlying mechanisms accounting for the relationship between attachment and obesity.

In this regard, the study of Bost and colleagues [28] represents an advance in this literature by showing that insecure attachment was linked children’s unhealthy food consumption indirectly through ineffective emotion regulation. Analyses suggested that insecure attachment may put caregivers at risk for using more unsupportive responses to children’s distress, which in turn may increase caregivers’ use of unresponsive feeding practices to children’s self-regulation of energy intake and put children at risk for developing overweight or obesity. These results also support a recent growing body of research suggesting that emotional responsiveness parallels feeding responsiveness [66,96,97,98].

Although the included studies in this review found associations between attachment quality and children’s weight status, the majority did not address the developmental mechanisms that underlie these associations, apart from the study of Bost and collaborators [28]. Therefore, future research should also examine the role of emotion processes and include other developmental factors implicated in caregiver–child interactions that also have a strong influence on the development of emotion regulation, such as the child’s temperament [99,100,101,102,103]. For example, caregivers may use food more often to comfort children with higher levels of reactivity (e.g., higher mood instability, sadness, anger, fear) and consequently children learn to eat in response to emotions [66,104]. Lastly, family routines (i.e., parental feeding practices, shared mealtime, screen time) should also be included, as they are important in the reduction of obesogenic behaviors [101,102], and the role of attachment and children’s self-regulation in these routines should be addressed.

Another limitation is that most studies’ samples did not include fathers. In the study of Keitel-Korndörfer and colleagues [29] none of the potential biological predictors (i.e., child’s BMI birth percentile and caregivers’ BMI) were significantly associated with children’s BMI percentiles, but mothers’ marital status was found to be related to child BMI; non-married mothers had children with higher BMI percentiles. Although more studies are needed, these data could suggest that fathers may play a protective role in child obesity risk. Future studies should include fathers and other caregivers in their samples to test these hypotheses. Furthermore, children’s attachment relationships to caregivers are independently co-constructed with the child and may therefore present specific or unique contributions to child outcomes [70,105,106]. The recent work of Fernandes and colleagues [107] revealed that the combined influence of mother–child and father–child attachment security on children’s emotion regulation was stronger than when considering the individual contributions of each parent. These findings highlight the importance of including father–child attachment when examining the role of emotion regulation in obesity risk, especially given the strong role of this developmental mechanism in eating behaviors (e.g., [35,37,38]).

In addition to having found few studies that address the relationship between attachment and obesity in preschool years, a great diversity of methodologies was observed, especially regarding to the sample and the instruments used, which makes it difficult to compare the results and allow a greater reliability in the interpretation of the results. For example, in the study conducted by Hepworth and collaborators [77], attachment measured using the AAS predicted children’s weight-for-length Z-scores, but not attachment assessed using the ECR. Importantly, there is also a small number of longitudinal studies examining attachment-obesity associations, which makes it difficult to draw conclusions about the temporal relation between attachment and obesity. Finally, according to systematic reviews’ goals and methods, the quality of studies and their size effects were not evaluated, limiting extrapolation to clinical designs. This decision was taken because there are few validated tools for the assessment of quality in observational studies [108,109].

Despite these limitations, by relying on a systematized approach, this review shows that attachment relationships have implications for the development of overweight and obesity as early as the preschool years, identifying important directions for future research. Studying the role of attachment quality in obesity during early development may inform prevention and intervention programs that focus on early modifiable behaviors that may halt unhealthy trajectories, which is important because obesity tracks from preschool to adolescence and into adulthood [10,11,110].

## Figures and Tables

**Table 1:** Summary of studies examining attachment quality and children’s weight status or food consumption.

Authors (Year) Country	Sample Characteristics	Study Design	Attachment Measures	Weight Status or Food Consumption Measures
Anderson and Whitaker (2011) (US) [24]	6650 children (female = 48.7%; male = 51.3%) and mothers	Longitudinal cohort	Toddler Attachment Sort-45 (assessed at 24 months)	BMI percentiles (assessed at 4^1^⁄_2_ years)
	Children’s age range = 24.3 to 54.3 months			
	Mothers’ age = ≥35 (17.5%), >30–35 (25.6%), >25–30 (25.7%), >20–25 (23.8%), 15–20 (7.3%)			
	Children’s race/ethnicity: White (55.9%), Black (15.4%), Hispanic (22.6%), other race (6.1%)			
	Maternal educational level: college graduate (26.4%), some college (26.7%), high school degree (28.9%), less than high school degree (18%)			
	Families’ income (income to poverty ratio): >3.00 (25.7%), 1.86–3.00 (28.1%), 1.00–1.85 (23.5%), 0.50–0.99 (12.7%), <0.50 (10%)			
	Caregivers’ marital status/living arrangements: living together (79.1%), separated (20.9%)			
Anderson et al. (2012) (US) [25]	977 children (female = 50.4%; male = 49.6%) and mothers	Longitudinal cohort	Strange Situation (assessed at 15 and 36 months) Attachment Q-set (assessed at 24 months)	BMI percentiles (assessed at 15 or 12 years)
	Children’s age range = 15 to 36 months			
	Mothers’s race/ethnicity: White (80.7%), nonwhite (19.3%)			
	Maternal educational level: graduate degree (15.7%), bachelor degree (22.4%), some college or associate degree (33.2%), high school degree (20.6%), less than high school degree (8.2%)			
	Families’ income (income to poverty ratio): ≥5.00 (23.2%), 3.00–4.99 (28.1%), 1.86–2.99 (22%), 1.00–1.85 (14.9%), <1.00 (11.9%)			
Bost et al. (2014) (US) [28]	497 caregivers (female = 90%; male = 10%) of children	Cross- sectional	Relationship Questionnaire	Early Childhood Longitudinal Study-B parent interview child health section
	Children’s age range = 2.5 to 3.5 years			
	Caregivers’ age mean = 32.5 years			
	Caregivers’ race/ethnicity: White (78%), African-American (18.2%), Asian (8%), Latino (3.8%)			
	Caregivers’ educational level: postgraduate degree (27.9%), college degree (26.3%), some college or technical training (32.1%), high school degree or less (11%)			
	Families’ income (annual household income): <40,000 (47.8%), <24,000 (29%)			
de Campora et al. (2019) (IT) [76]	51 mothers of children (female = 43.4%; male = 56.6%)	Longitudinal birth cohort	Reflective Functioning Scale of the Adult Attachment Interview (assessed during pregnancy)	BMI (assessed at 3 years)
	Children’s age range = birth to 3 years			
	Mothers’ age mean = 34.8 years			
	Maternal educational level: bachelor degree or more (43.1%), high school degree or less (56.9%)			
Hepworth et al. (2021) (US) [77]	55 mothers of children (female = 46%; male = 54%)	Cross- sectional	Adult Attachment Scale Experiences in Close Relationships Scale	Weight-for-length Z-scores
	Children’s age mean = 12.7 months			
	Mothers’ age mean = 30.9 years			
	Mothers’ race/ethnicity: Latino (95%)			
	Maternal educational level: high school degree (51%), less than high school degree (49%)			
	Maternal professional situation: employed (53%), unemployed (47%)			
	Families’ income (status): low			
	Maternal marital status/living arrangements: married or cohabitating (82%)			
Keitel-Korndörfer et al. (2015) (DE) [29]	62 children (female = 56%; male = 44%) and mothers (normal-weight = 50%; obese = 50%)	Cross- sectional	Attachment Q-Set	BMI percentiles
	Children’s age range = 19 to 58 months			
	Mothers’ age range = 22.8 to 44.0 years			
	Maternal educational level: high school degree (normal-weight = 57%; obese = 48%), less than high school degree (normal-weight = 43%; obese = 52%)			
	Maternal marital status/living arrangements: in a relationship with the father (normal-weight = 81%; obese = 71%), not in relationship (normal-weight = 19%; obese = 29%)			
Keitel-Korndörfer et al. (2016) (DE) [78]	60 children (female = 55%; male = 45%) and mothers (normal-weight = 50%; obese = 50%)	Cross- sectional	Reflective Functioning Scale of the Adult Attachment Interview Attachment Q-Set	BMI percentiles
	Children’s age range = 18 to 55 months			
	Mothers’ age mean = 31.7 years			
	Maternal educational level: high school degree (normal-weight = 55%; obese = 50%), less than high school degree (normal-weight = 45%; obese = 50%)			
	Maternal marital status/living arrangements: in a relationship with the father (normal-weight = 80%; obese = 70%), not in relationship (normal-weight = 20%; obese = 30%)			
Stenhammar et al. (2010) (SE) [72]	873 children and caregivers (mothers = 865; fathers = 746)	Cross- sectional	Relationship Questionnaire	BMI percentiles
	Children’s age = 3 years			
	Caregivers’ educational level: college degree (48.2%), some college (2.8%), high school degree (42.8%), less than high school degree (6.2%)			
	Caregivers’ professional situation: employed (77%), parental leave (11%), student (7%), unemployed (5%)			
	Caregivers’ marital status/living arrangements: living together (92.9%), separated (7.1%)			

**Table 2:** Summary of key findings on the relationship between caregiver attachment and obesity.

Authors (Year) Country	Key Findings	Covariates
Bost et al. (2014) (US) [28]	Caregivers’ insecure attachment was associated with children’s unhealthy food consumption indirectly through unsupportive responses (punishing or dismissing responses) to children’s distress. These unsupportive responses predicted the increased use of emotion-related and pressuring feeding styles.	Controlled for children’s and caregivers’ age, children’s gender, caregivers’ BMI, race/ethnicity, education level, depression, and anxiety.
de Campora et al. (2019) (IT) [76]	Maternal mentalization was only marginally significantly associated with children’s BMI. Multiple regression models suggest that maternal mentalization might explain the variance of children’s BMI beyond the effects of maternal emotional dysregulation.	No information available.
Hepworth et al. (2021) (US) [77]	Mother’s insecure attachment style, according to the AAS, was associated with children’s higher weight-for-length Z-scores. This association was not mediated by maternal sensitivity. Mother’s attachment avoidance and attachment anxiety, according to the ECR, did not predicted children’s weight-for-length Z-scores.	Controlled for children’s age at baseline, children’s gender, maternal BMI, family cumulative risk, and randomized controlled trial (RCT) group.
Keitel-Korndörfer et al. (2016) (DE) [78]	Lower maternal mentalization was associated with children’s higher BMI percentiles indirectly through a lower quality of mother–child attachment.	Controlled for maternal intelligence quotient (IQ) and maternal depression.
Stenhammar et al. (2010) (SE) [72]	Caregivers’ insecure attachment was associated with overweight in children. This association was not independent of maternal stress. Maternal stress was associated with overweight and underweight in children.	Controlled for children’s and caregivers’ gender, children’s number of older and younger siblings, mothers’ self-perceived weight, caregivers’ age, education level, professional situation, and living arrangements.

**Table 3:** Summary of key findings on the relationship between caregiver–child attachment and obesity.

Authors (Year) Country	Key Findings	Covariates
Anderson and Whitaker (2011) (US) [24]	The odds for obesity at 4^1^⁄_2_ years of age were 1.30 times higher for children with an insecure mother–child attachment than for children with a secure mother–child attachment measured at 24 months.	Controlled for children’s and mothers’ age, children’s gender, children’s birth weight, maternal BMI, race/ethnicity, education level, income to poverty ratio, the quality of mother–child interaction during play (maternal responsiveness, child engagement, and child negativity), and parenting practices related to obesity (television/video/DVD viewing and the frequency of eating family dinners).
Anderson et al. (2012) (US) [25]	Insecure mother–child attachment at 24 months of age (according to the AQS) was associated with increased odds for adolescent obesity, but not at 15 and 36 months (according to the SSP). The odds for adolescent obesity were 2.45 times higher for children with an insecure mother–child attachment than for children with a secure mother–child attachment.	Controlled for children’s age, children’s birth weight, maternal BMI, race/ethnicity, education level, and income to poverty ratio.
Keitel-Korndörfer et al. (2015) (DE) [29]	Insecure mother–child attachment was associated with children’s higher BMI percentiles.	Controlled for children’s BMI birth percentile, caregivers’ BMI, and maternal marital status.
Keitel-Korndörfer et al. (2016) (DE) [78]	Lower maternal mentalization was associated with children’s higher BMI percentile indirectly through a lower quality of mother–child attachment.	Controlled for maternal intelligence quotient (IQ) and maternal depression.

## Data Availability

Data sharing is not applicable to this article as no new data were created or analysed in this study.
